# Mechanical thrombectomy in nonagenarians: real-world outcomes from a highly selected cohort treated at a comprehensive stroke center

**DOI:** 10.1186/s12883-026-05253-1

**Published:** 2026-08-07

**Authors:** Bartosz Jabłoński, Adam Wyszomirski, Aleksandra Pracoń, Marcin Stańczak, Anna Gwiazda, Łukasz Maciołek, Barbara Kołodziej-Czubacka, Edyta Szurowska, Dariusz Gąsecki, Bartosz Karaszewski

**Affiliations:** 1 Department of Adult Neurology, Faculty of Medicine, Medical University of Gdańsk, Gdańsk, Poland; 2 Department of Adult Neurology, University Clinical Centre, Gdańsk, Poland; 3 2nd Department of Radiology, Faculty of Health Sciences with the Institute of Maritime and Tropical Medicine, Medical University of Gdańsk, Gdańsk, Poland; 4 Department of Radiology, University Clinical Centre, Gdańsk, Poland; 5 Translational Brain Diseases Centre, The Fahrenheit Union of Universities, Gdańsk, Poland

**Keywords:** Mechanical thrombectomy, Nonagenarians, Acute ischemic stroke, Hemorrhagic transformation, Very elderly

## Abstract

**Background:**

Evidence supporting mechanical thrombectomy (MT) in very elderly patients remains limited, as nonagenarians are underrepresented in randomized trials and frequently excluded from MT in routine practice. We evaluated the effectiveness and safety of MT in carefully selected patients aged ≥ 90 years treated at a comprehensive stroke center.

**Methods:**

We conducted a retrospective single-center cohort study including consecutive patients with anterior circulation large vessel occlusion treated with MT. Fifty nonagenarians treated between 2017 and 2025 were compared with 100 younger patients treated in 2023. In both groups, only patients with preserved pre-stroke functional independence (modified Rankin Scale [mRS] 0–2) were included. Nonagenarians underwent detailed pre-treatment clinical assessment prior to treatment. Primary outcomes were functional independence (mRS 0–2) and all-cause mortality at 90 days. Secondary outcomes included successful reperfusion (mTICI 2b–3), neurological status at discharge, and hemorrhagic complications. Multivariable regression models were used to adjust for baseline imbalances and clinically relevant prognostic factors.

**Results:**

Nonagenarians had a substantially higher burden of comorbidities and worse pre-stroke functional status than younger patients. Successful reperfusion rates were similar between groups (92% vs. 90%). Functional independence at 90 days was achieved in 18% of nonagenarians and 50% of younger patients, while 90-day mortality was 52% and 20%, respectively. In unadjusted analyses, nonagenarians had lower odds of functional independence and higher odds of mortality. After adjustment for baseline clinical and prognostic factors, the estimated associations were attenuated, although confidence intervals remained wide and the study could not exclude a clinically relevant age-associated effect. Parenchymal hematoma occurred more frequently among nonagenarians, whereas rates of symptomatic intracerebral hemorrhage were comparable between groups.

**Conclusions:**

In this highly selected real-world cohort, MT achieved high rates of successful reperfusion in nonagenarians, but functional outcomes and mortality were substantially less favorable than in younger patients. Adjustment for baseline clinical and prognostic factors attenuated the estimated associations, although clinically relevant age-associated differences could not be excluded. These findings support cautious, individualized treatment decisions incorporating chronological age, pre-stroke function, comorbidity burden, imaging findings, and the overall clinical profile.

## Background

Mechanical thrombectomy (MT) is the standard treatment for acute ischemic stroke (AIS) caused by large vessel occlusion. The HERMES meta-analysis established the benefit of MT by pooling individual patient data from five pivotal randomized controlled trials and demonstrated significantly reduced disability at 90 days compared with best medical therapy alone [1–6]. Importantly, treatment effects favoring MT were observed across multiple predefined subgroups, including elderly patients aged ≥ 80 years [4]. However, individuals of very advanced age remained markedly underrepresented in these trials, and patients aged ≥ 90 years constituted only a marginal proportion of the study populations.

Subsequent analyses have suggested that advanced age alone should not be regarded as an absolute contraindication to thrombectomy. A dedicated HERMES substudy focusing on patients aged ≥ 85 years demonstrated that, despite higher mortality and worse functional outcomes than younger individuals, elderly patients continued to derive substantial benefit from MT [7]. Likewise, expansion of thrombectomy indications through DAWN and DEFUSE-3 extended treatment eligibility to selected patients presenting up to 24 h from symptom onset [8, 9], while the AURORA meta-analysis demonstrated consistent treatment effects across age strata without evidence of age-related heterogeneity [10]. Nevertheless, nonagenarians remained only sparsely represented in these studies.

As life expectancy continues to increase worldwide [11], the number of nonagenarians presenting with AIS is steadily growing. Clinical decision-making in this population remains challenging because chronological age is frequently accompanied by frailty, reduced physiological reserve, multiple comorbidities, cognitive impairment, and limited recovery potential [12]. Consequently, concerns regarding treatment futility and procedural risk often influence eligibility for MT in routine clinical practice [13, 14].

Available observational evidence suggests that MT can be technically successful in nonagenarians, with recanalization rates comparable to those observed in younger patients. However, functional outcomes and mortality vary substantially across published cohorts [15, 16]. Importantly, this heterogeneity likely reflects differences in baseline functional status, comorbidity burden, local treatment practices, and patient selection strategies. Therefore, outcomes reported in nonagenarians may depend not only on chronological age itself but also on how patients are selected for intervention.

Despite the central role of patient selection in contemporary stroke care, outcomes among carefully selected nonagenarians treated at experienced comprehensive stroke centers remain insufficiently characterized. Therefore, the aim of the present study was to evaluate the effectiveness and safety of MT in carefully selected patients aged ≥ 90 years treated at a single comprehensive stroke center. To provide clinical context for the observed outcomes, results were additionally compared with those of a younger contemporary cohort undergoing thrombectomy during routine clinical practice.

## Materials and methods

### Study design and setting

This was a retrospective, observational cohort study conducted at a single tertiary comprehensive stroke center. The study evaluated the effectiveness and safety of MT in carefully selected patients aged ≥ 90 years and compared the observed outcomes with those of a younger contemporary cohort. Consecutive patients treated with MT for AIS were identified using the hospital information system of the University Clinical Center in Gdańsk, Poland. All included patients were managed within the Stroke Unit, Department of Adult Neurology, University Clinical Center in Gdańsk, Poland. The study period covered January 2017 to April 2025.

### Study population

Patients were categorized according to age at stroke onset into a nonagenarian group (≥ 90 years) and a younger comparison group (< 90 years).

The nonagenarian cohort comprised all consecutive patients aged ≥ 90 years who underwent MT between January 2017 and April 2025 and fulfilled the predefined eligibility criteria.

To provide contemporary clinical context, a comparator cohort consisting of consecutive patients aged < 90 years treated during a recent period of stable institutional thrombectomy practice was identified. This cohort comprised patients treated between January and July 2023, a period reflecting contemporary endovascular techniques, established treatment workflows, and uniform standards of stroke care.

For both groups, inclusion required documented pre-stroke functional independence (modified Rankin Scale score 0–2) [17], known symptom onset time, and anterior circulation large vessel occlusion treated with MT.

Because nonagenarians were accrued over a longer period and underwent a dedicated pre-treatment clinical assessment, whereas the younger cohort represented routine contemporary clinical practice, the two groups were not intended to represent fully exchangeable populations. Therefore, the younger cohort was included primarily to provide clinical context for the observed outcomes among nonagenarians rather than to support direct age-based causal comparisons.

### Eligibility criteria

Eligibility for MT required the presence of a large vessel occlusion in the anterior circulation, involving the intracranial internal carotid artery (ICA), the M1 segment of the middle cerebral artery, or a proximal M2 segment deemed suitable for MT. Neuroimaging criteria included an Alberta Stroke Program Early CT Score (ASPECTS) > 5.

All patients underwent MT according to the standard institutional workflow. Qualification was generally performed within the conventional early therapeutic window and did not require routine use of advanced perfusion-based imaging techniques. Treatment decisions were made irrespective of prior administration of intravenous thrombolysis. A clinically significant neurological deficit, as determined by the treating stroke neurologist, was required for intervention.

All MT procedures were performed by experienced neurointerventionalists according to institutional practice. Stent retriever thrombectomy, either alone or combined with aspiration, was the predominant procedural technique throughout the study period, whereas aspiration-first approaches were used less frequently.

### Clinical assessment of nonagenarians

In patients aged ≥ 90 years, eligibility for MT was determined through a structured pre-treatment clinical assessment aimed at identifying individuals with a realistic potential for benefit while minimizing futile treatment.

Each case was reviewed by a stroke specialist with more than 10 years of experience in acute stroke management and thrombectomy decision-making. Particular emphasis was placed on accurate determination of pre-stroke functional status. The pre-stroke mRS was established through detailed review of medical records and, when necessary, supplemented by structured telephone interviews with family members or caregivers.

Comorbidity burden was systematically evaluated, with special attention to conditions associated with limited recovery potential. Patients with end-stage renal failure or other terminal comorbidities were excluded. An absence of terminal comorbidities and a reasonable expectation of survival beyond the acute stroke period were considered during clinical decision-making.

Time-from-onset criteria were also carefully considered. The majority of nonagenarian patients (45 of 50) were treated within 7.5 h from symptom onset, corresponding to the conventional early treatment window. Only a minority were treated beyond this timeframe. Two patients were treated according to DAWN eligibility criteria, two based on favorable collateral circulation status, and one fulfilled perfusion mismatch criteria consistent with DEFUSE-3. The median time from symptom onset to groin puncture was 290 (interquartile range 218–370) minutes.

The goal of this assessment was not solely to maximize the likelihood of functional independence, but rather to avoid futile treatment in patients unlikely to derive meaningful clinical benefit from thrombectomy.

### Imaging protocol

The standard imaging workflow included non-contrast computed tomography (CT) to exclude intracranial hemorrhage and computed tomography angiography (CTA) to confirm large vessel occlusion. Computed tomography perfusion (CTP) or magnetic resonance imaging (MRI) with diffusion-weighted imaging and magnetic resonance angiography were performed selectively when clinically indicated.

### Outcomes

The primary outcomes were functional independence at 90 days, defined as a modified Rankin Scale (mRS) score of 0–2, and all-cause mortality at 90 days. Functional outcomes were assessed using standardized telephone interviews conducted by trained clinicians or research personnel with patients or their caregivers.

Secondary outcomes included successful reperfusion, defined as a modified Thrombolysis in Cerebral Infarction (mTICI) [18] score of 2b–3, neurological status at hospital discharge assessed using the National Institutes of Health Stroke Scale (NIHSS) [19], and hemorrhagic complications.

Hemorrhagic complications included parenchymal hematoma type 1 (PH1) and type 2 (PH2), classified according to the European Cooperative Acute Stroke Study (ECASS) criteria [20]. Symptomatic intracerebral hemorrhage (sICH) was defined according to the Safe Implementation of Thrombolysis in Stroke-Monitoring Study (SITS-MOST) criteria as a PH2 associated with neurological deterioration of ≥ 4 NIHSS points or leading to death. As an additional safety analysis, sICH was also assessed according to the Heidelberg Bleeding Classification criteria [21].

### Statistical analysis

The sample size was determined a priori based on functional independence (mRS 0–2) at 90 days, which was considered the primary efficacy outcome for sample size planning. Assuming event rates of 24% among nonagenarians and 49% among younger patients, derived from the HERMES elderly subgroup analysis [7], a two-sided alpha level of 0.05, 80% power, and a 1:2 allocation ratio, the required sample size was estimated at 44 patients in the nonagenarian group and 88 patients in the younger cohort. To account for potential uncertainty, the final sample sizes were increased to 50 and 100 patients, respectively. This calculation was intended for the unadjusted comparison of functional independence and was not designed to ensure power for adjusted analyses.

Continuous variables are presented as medians with interquartile ranges (IQRs), whereas categorical variables are reported as frequencies and percentages. Baseline differences between groups were assessed using quantile regression (τ = 0.5) for continuous variables and Fisher’s exact test for categorical variables.

Clinical outcomes were analyzed using generalized linear models (GLMs). Binary outcomes were modeled using logistic regression models with age group as the main explanatory variable, and results are presented as odds ratios (ORs) with 95% confidence intervals (CIs). For all binary outcomes, ORs compare nonagenarians with the younger cohort. NIHSS score at discharge was analyzed after log(NIHSS + 1) transformation to account for right-skewness and zero values, and results were presented as geometric mean ratios (GMRs), with values above 1 indicating higher discharge NIHSS scores among nonagenarians.

Given the substantial baseline differences between age groups and the presence of clinically relevant prognostic factors, primary adjusted analyses were performed using prespecified multivariable regression models. Covariates were selected a priori based on clinical relevance, established prognostic importance in acute ischemic stroke, baseline imbalance between groups, and model parsimony. To reduce the risk of overfitting given the limited sample size, the number of adjustment variables was intentionally restricted to a small set of prespecified covariates. Primary outcomes and NIHSS at discharge were adjusted for pre-stroke mRS, admission NIHSS score, ASPECTS score, onset-to-groin puncture time, and chronic kidney disease. The PH 1–2 outcome was adjusted for admission NIHSS score and intravenous thrombolysis. mTICI 2b–3 and sICH outcomes were analyzed only in unadjusted models because of the limited number of unsuccessful reperfusion and sICH events, respectively.

As an exploratory sensitivity analysis, propensity score overlap weighting was performed to target the average treatment effect in the overlap population (ATO). The propensity score model included admission NIHSS score, smoking status, pre-stroke mRS score, OCSP classification, prior antithrombotic therapy, onset-to-groin puncture time, ASPECTS score, dyslipidemia, hypertension, diabetes mellitus, atrial fibrillation, TOAST classification, chronic kidney disease, and intravenous thrombolysis. The ATO estimand corresponds to patients with baseline characteristics compatible with either age group and should not be interpreted as the average effect in the entire cohort. Covariate balance was assessed using standardized mean differences (SMDs), with values < 0.10 considered acceptable. Robust standard errors were used in weighted analyses.

For the two prespecified primary outcomes, p-values from the primary adjusted regression models were adjusted using the Benjamini–Hochberg false discovery rate procedure. P-values from unadjusted analyses, secondary outcomes, and sensitivity analyses were not adjusted for multiplicity and were interpreted as exploratory. Confidence intervals were not adjusted for multiplicity. Statistical significance for the primary adjusted analysis was defined as a two-sided FDR-adjusted p-value < 0.05.

Missing data were not imputed because only one discharge NIHSS value was missing. All analyses were performed using R version 4.4.2 (R Foundation for Statistical Computing, Vienna, Austria).

### Ethical considerations

The study was approved by the local Ethics Committee of the Medical University of Gdańsk (approval number: KB/87/2026, approved on February 23, 2026). Due to the retrospective nature of the study, the requirement for informed consent was waived. The study was conducted and reported in accordance with the Strengthening the Reporting of Observational Studies in Epidemiology (STROBE) guidelines for cohort studies.

## Results

### Baseline characteristics

**Fig. 1:**
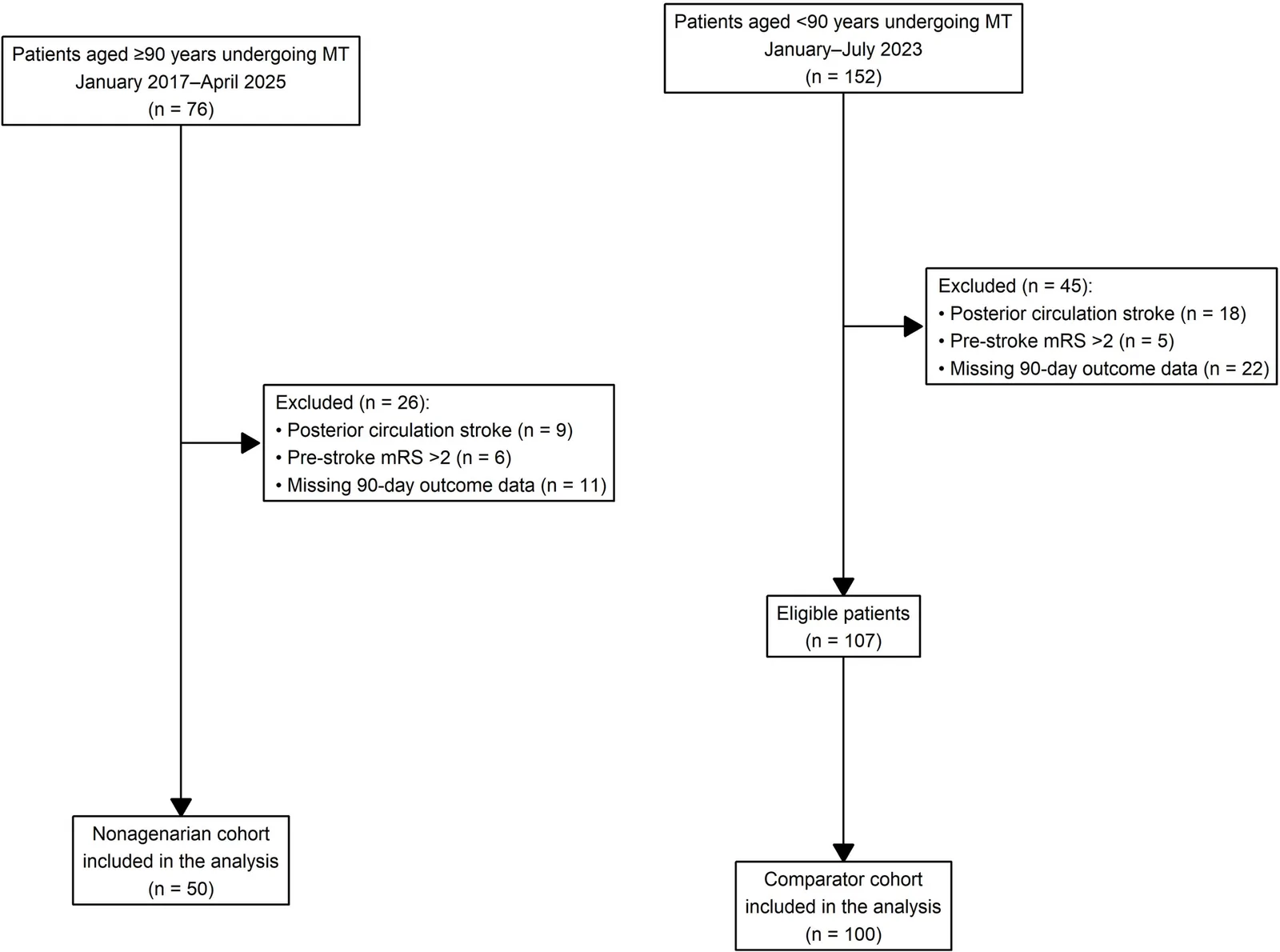
Study flowchart illustrating patient selection for the nonagenarian and younger comparator cohorts

A total of 150 patients with acute ischemic stroke treated with MT were included in the study, comprising 50 nonagenarians and 100 younger patients. The patient selection flow chart is provided in Fig. 1, and baseline characteristics are presented in Table 1.

**Table 1:** Baseline characteristics

Variable^1^	Nonagenarians (*n* = 50)	Younger cohort (*n* = 100)	*P*-value^1^
Age [years]			< 0.001
Median (Q1–Q3)	91 (90–93)	70 (65–76)	
Female, *n* / *N* (%)	37 / 50 (74)	57 / 100 (57)	0.050
Atherosclerosis, *n* / *N* (%)	12 / 50 (24)	40 / 100 (40)	0.069
Smoking, *n* / *N* (%)	7 / 50 (14)	45 / 100 (45)	< 0.001
pre mRS, *n* / *N* (%)			< 0.001
0	4 / 50 (8)	54 / 100 (54)	
1	19 / 50 (38)	38 / 100 (38)	
2	27 / 50 (54)	8 / 100 (8)	
NIHSS at admission			0.117
Median (Q1–Q3)	18.5 (15–22)	16 (11–19)	
OCSP, *n* / *N* (%)			0.307
LACS	0 / 50 (0)	1 / 100 (1)	
PACS	7 / 50 (14)	23 / 100 (23)	
TACS	43 / 50 (86)	76 / 100 (76)	
Intravenous thrombolysis, *n* / *N* (%)	31 / 50 (62)	67 / 100 (67)	0.587
Antithrombotics before onset, *n* / *N* (%)			0.937
Single antiplatelet	12 / 50 (24)	27 / 100 (27)	
DAPT	1 / 50 (2)	3 / 100 (3)	
DOAC	6 / 50 (12)	11 / 100 (11)	
VKA	2 / 50 (4)	2 / 100 (2)	
None	29 / 50 (58)	57 / 100 (57)	
Time to MT			0.722
Median (Q1–Q3)	292.5 (208–360)	290 (221–376.5)	
ASPECTS, *n* / *N* (%)			0.556
6	0 / 50 (0)	5 / 100 (5)	
7	4 / 50 (8)	8 / 100 (8)	
8	5 / 50 (10)	15 / 100 (15)	
9	14 / 50 (28)	21 / 100 (21)	
10	27 / 50 (54)	51 / 100 (51)	
Site of occlusion, *n* / *N* (%)			0.146
carotidL	1 / 50 (2)	1 / 100 (1)	
carotidT	4 / 50 (8)	6 / 100 (6)	
ICA	7 / 50 (14)	8 / 100 (8)	
ICA+M1	2 / 50 (4)	20 / 100 (20)	
ICA+M2	2 / 50 (4)	5 / 100 (5)	
M1	23 / 50 (46)	39 / 100 (39)	
M2	11 / 50 (22)	21 / 100 (21)	
Dyslipidemia, *n* / *N* (%)	28 / 50 (56)	82 / 100 (82)	0.001
Hypertension, *n* / *N* (%)	45 / 50 (90)	85 / 100 (85)	0.456
Diabetes mellitus, *n* / *N* (%)	15 / 50 (30)	27 / 100 (27)	0.704
Atrial fibrillation, *n* / *N* (%)	35 / 50 (70)	41 / 100 (41)	< 0.001
Chronic kidney disease, *n* / *N* (%)	40 / 50 (80)	24 / 100 (24)	< 0.001
TOAST, *n* / *N* (%)			0.156
CE	29 / 50 (58)	41 / 100 (41)	
LAA	9 / 50 (18)	28 / 100 (28)	
Undetermined etiology	12 / 50 (24)	27 / 100 (27)	
Other determined etiology	0 / 50 (0)	4 / 100 (4)	

*mRS* Modified Rankin Scale, *NIHSS* National Institutes of Health Stroke Scale, *OCSP* Oxfordshire Community Stroke Project, *LACS* Lacunar Syndrome, *PACS* Partial Anterior Circulation Stroke Syndrome, *TACS* Total Anterior Circulation Stroke, *DAPT* Dual Antiplatelet Therapy, *DOAC* Direct Oral Anticoagulant, *VKA* Vitamin K Antagonist, *MT* Mechanical thrombectomy, *ASPECTS* Alberta Stroke Program Early CT Score, *ICA* Internal Carotid Artery, *M1* Sphenoidal segment (stem), *M2* Insular segment, *TOAST* Trial of ORG 10,172 in Acute Stroke Treatment, *CE* Cardioembolism, *LAA* Large-Artery Atherosclerosis, *Q1* First quartile, *Q3* Third quartile

^1^Quantile regression with tau = 0.5 (median) for numerical variable and Fisher’s exact test for categorical variable

Compared with the younger patients, nonagenarians had a less favorable baseline clinical profile. Pre-stroke disability was present in 92% of nonagenarians compared with 46% of younger patients (*P* < 0.001). Nonagenarians also had higher rates of atrial fibrillation (70% vs. 41%, *P* < 0.001) and chronic kidney disease (80% vs. 24%, *P* < 0.001), whereas smoking and dyslipidemia were more common in younger patients. Stroke severity, imaging characteristics, and treatment-related variables were broadly comparable between groups, including admission NIHSS, onset-to-groin puncture time, ASPECTS distribution, occlusion site, and intravenous thrombolysis rates.

### Outcomes among nonagenarians

Among nonagenarians, successful reperfusion defined as mTICI 2b–3 was achieved in 46 patients (92%). Functional independence at 90 days (mRS 0–2) occurred in 9 patients (18%), whereas 26 patients (52%) died within 90 days. Parenchymal hematoma occurred in 14 patients (28%), and symptomatic intracerebral hemorrhage was observed in 3 patients (6%) according to SITS-MOST criteria and in 5 patients (10%) according to the Heidelberg Bleeding Classification. The main outcome results are summarized in Table 2.

**Table 2:** Study outcomes

Outcome	Age		Unadjusted regression		Adjusted regression	
	Nonagenarians (*n* = 50)	Younger cohort (*n* = 100)	Odds ratio (95% CI)	*P*-value	Odds ratio (95% CI)	*P*-value
mRS 0‒2 at 90 days, *n* / *N* (%)	9 / 50 (18)	50 / 100 (50)	0.22 (0.09, 0.48)	< 0.001	0.38 (0.11, 1.19)	0.210
NIHSS discharge, Geometric Mean (GSD); *N* missing	13.6 (2.8); 0	6.4 (3.2); 1	2.12 (1.45, 3.10)*	< 0.001	1.37 (0.86, 2.18)*	0.186
mTICI 2b‒3, *n* / *N* (%)	46 / 50 (92)	90 / 100 (90)	1.28 (0.40, 4.86)	0.692	-	-
Mortality at 90 days, *n* / *N* (%)	26 / 50 (52)	20 / 100 (20)	4.33 (2.09, 9.22)	< 0.001	1.73 (0.59, 5.13)	0.316
Parenchymal hematoma (PH 1‒2), *n* / *N* (%)	14 / 50 (28)	10 / 100 (10)	3.50 (1.44, 8.82)	0.006	3.18 (1.28, 8.15)	0.014
sICH SITS-MOST, *n* / *N* (%)	3 / 50 (6)	4 / 100 (4)	1.53 (0.29, 7.22)	0.587	-	-
sICH Heidelberg classification, *n* / *N* (%)	5 / 50 (10)	11 / 100 (11)	0.90 (0.27, 2.63)	0.852	-	-

*mRS* Modified Rankin Scale, *NIHSS* National Institutes of Health Stroke Scale, *mTICI* modified Thrombolysis in Cerebral Infarction, *PH* Parenchymal Hemorrhage, *sICH* Symptomatic Intracerebral Hemorrhage, *CI* Confidence Interval, *GMR* Geometric Mean Ratio, *GSD* Geometric Standard Deviation, *IPW* Inverse Probability Weighting

*Results presented as GMR (95% CI)

### Comparative clinical outcomes

In unadjusted analyses, nonagenarians had lower odds of functional independence at 90 days than younger patients (18% vs. 50%; OR 0.22, 95% CI 0.09–0.48; *P* < 0.001) and higher odds of 90-day mortality (52% vs. 20%; OR 4.33, 95% CI 2.09–9.22; *P* < 0.001). After adjustment for pre-stroke mRS, admission NIHSS score, ASPECTS score, onset-to-groin puncture time, and chronic kidney disease, the estimated associations were attenuated and remained imprecise: adjusted OR 0.38 (95% CI 0.11–1.19; FDR-adjusted *P* = 0.210) for functional independence and adjusted OR 1.73 (95% CI 0.59–5.13; FDR-adjusted *P* = 0.316) for mortality.

Successful reperfusion rates were high and comparable between groups (92% vs. 90%; OR 1.28, 95% CI 0.40–4.86; *P* = 0.692). This endpoint was analyzed only in the unadjusted model because of the limited number of unsuccessful reperfusion events. Nonagenarians had higher discharge NIHSS in the unadjusted analysis (GMR 2.12, 95% CI 1.45–3.10; *P* < 0.001), but this difference was attenuated after adjustment and was no longer statistically significant (adjusted GMR 1.37, 95% CI 0.86–2.18; *P* = 0.186).

### Safety outcomes

Parenchymal hematoma occurred more frequently among nonagenarians than in younger patients (28% vs. 10%; OR 3.50, 95% CI 1.44–8.82; *P* = 0.006). This association persisted after adjustment for admission NIHSS score and intravenous thrombolysis (adjusted OR 3.18, 95% CI 1.28–8.15; *P* = 0.014).

Rates of symptomatic intracerebral hemorrhage were comparable between groups according to both definitions. According to SITS-MOST criteria, sICH occurred in 6% of nonagenarians and 4% of younger patients (OR 1.53, 95% CI 0.29–7.22; *P* = 0.587). According to the Heidelberg Bleeding Classification, sICH occurred in 10% and 11% of patients, respectively (OR 0.90, 95% CI 0.27–2.63; *P* = 0.852). These outcomes were analyzed only in unadjusted models because of the limited number of sICH events.

### Sensitivity analysis using propensity score overlap weighting

**Fig. 2:**
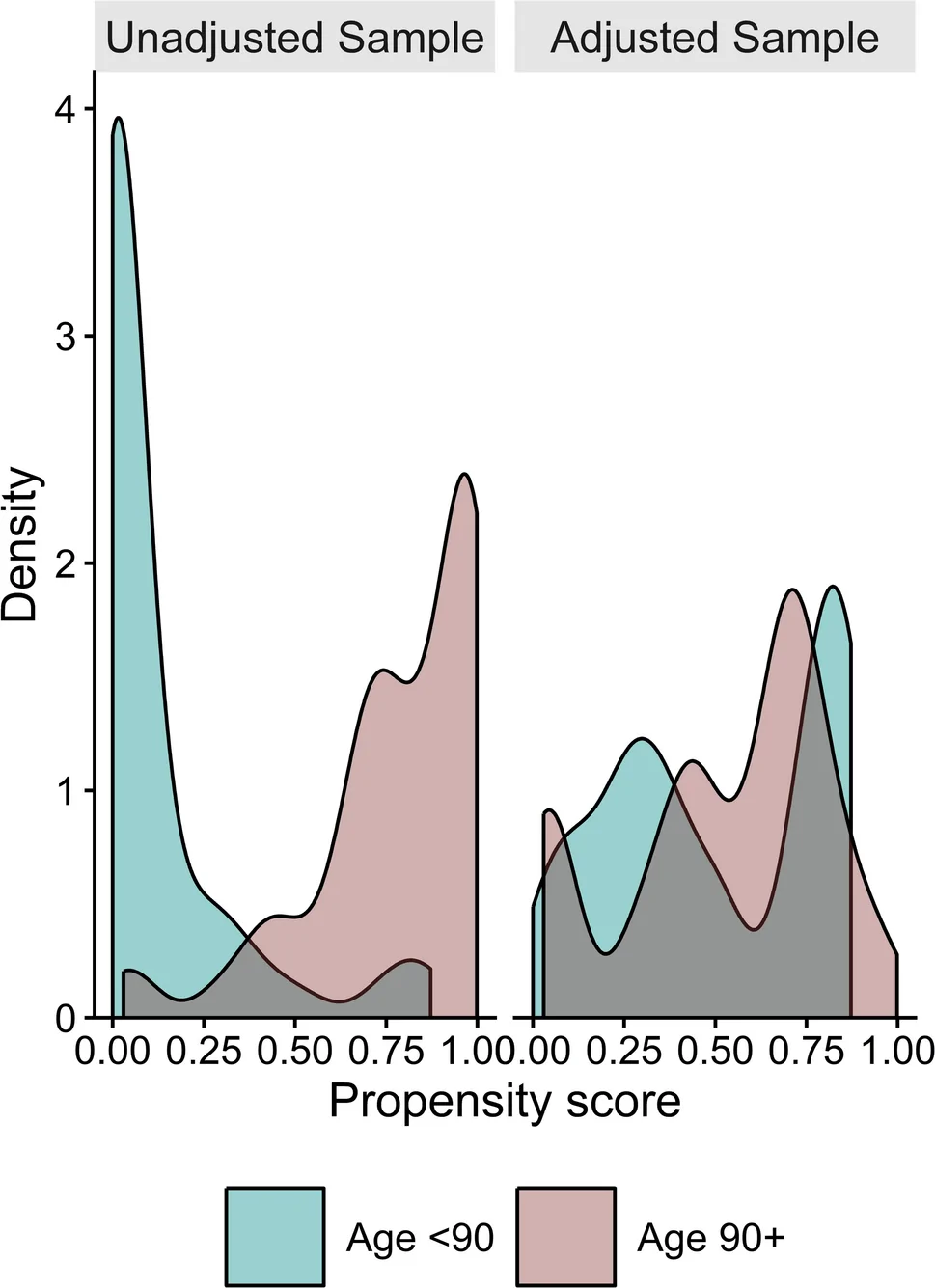
Propensity score distributions before and after weighting

**Fig. 3:**
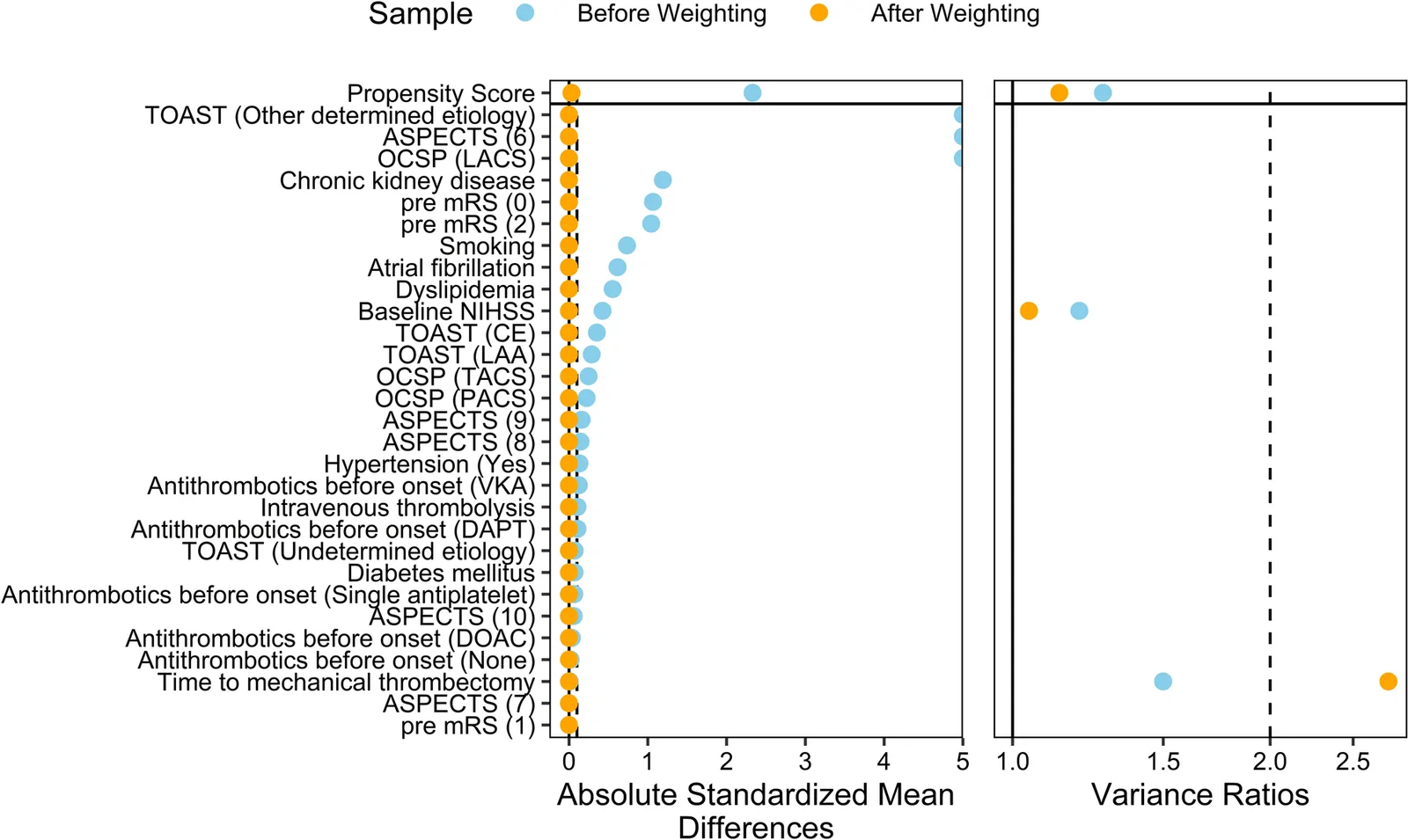
Covariate balance before and after weighting

Propensity score overlap weighting was performed as an exploratory sensitivity analysis targeting the average treatment effect in the overlap population. Propensity score distributions before and after weighting are shown in Fig. 2, and covariate balance is shown in Fig. 3. In this analysis, nonagenarians had lower odds of functional independence at 90 days (OR 0.19, 95% CI 0.04–0.89; *P* = 0.035), whereas the association with 90-day mortality remained imprecise and was not statistically significant (OR 1.75, 95% CI 0.52–5.85; *P* = 0.363).

For secondary and safety outcomes, overlap-weighted estimates suggested higher discharge NIHSS and higher odds of parenchymal hematoma among nonagenarians, while estimates for successful reperfusion and symptomatic intracerebral hemorrhage were imprecise, with wide confidence intervals. Full sensitivity analysis results are presented in Table 3.

**Table 3:** Sensitivity outcomes analysis

	IPW regression	
Outcome	Odds ratio (95% CI)	*P*-value
mRS 0‒2 at 90 days	0.19 (0.04, 0.89)	0.035
NIHSS discharge	1.78 (1.05, 3.02)*	0.033
mTICI 2b‒3	4.14 (0.70, 24.5)	0.116
Mortality at 90 days	1.75 (0.52, 5.85)	0.363
Parenchymal hematoma (PH 1‒2)	5.79 (1.18, 28.4)	0.031
sICH SITS-MOST	1.82 (0.16, 21.4)	0.630
sICH Heidelberg classification	1.27 (0.19, 8.65)	0.804

*mRS* Modified Rankin Scale, *NIHSS* National Institutes of Health Stroke Scale, *mTICI* modified Thrombolysis in Cerebral Infarction, *PH* Parenchymal Hemorrhage, *sICH* Symptomatic Intracerebral Hemorrhage, *CI* Confidence Interval, *GMR* Geometric Mean Ratio, *IPW* Inverse Probability Weighting

*Results presented as GMR (95% CI)

## Discussion

In this single-center cohort study, we evaluated the effectiveness and safety of MT in a consecutive cohort of eligible nonagenarians treated at a comprehensive stroke center. The principal findings were threefold. First, successful reperfusion rates remained high in patients aged ≥ 90 years and were comparable to those observed in younger patients. Second, despite excellent technical success, nonagenarians experienced substantially worse crude functional outcomes and higher mortality, with more than half of the patients dying within 90 days. Third, adjustment for baseline characteristics and prognostic factors attenuated the associations between nonagenarian status and the two primary outcomes, although the confidence intervals remained wide and did not exclude clinically important age-associated differences.

The efficacy of MT in elderly patients has been established through randomized evidence, although very elderly individuals remain underrepresented in pivotal trials. In the HERMES collaboration, patients aged ≥ 80 years derived significant benefit from thrombectomy despite worse absolute outcomes compared with younger individuals [1]. Similarly, a dedicated HERMES analysis focusing on patients aged ≥ 85 years demonstrated persistent treatment benefit despite increased mortality and disability [7]. Nevertheless, nonagenarians represented only a small fraction of these trial populations, limiting the direct applicability of randomized evidence to this rapidly growing age group.

Our findings are broadly consistent with previously published observational data. In a systematic review and meta-analysis including 657 nonagenarians, Bai et al. reported pooled rates of functional independence of approximately 22% and three-month mortality approaching 44%, despite high rates of successful recanalization [15]. Similarly, Zarrintan et al., in a meta-analysis of 47 studies, demonstrated substantially worse functional outcomes and higher mortality among nonagenarians compared with younger patients, while rates of successful reperfusion and symptomatic intracerebral hemorrhage remained largely comparable across age groups [16]. The outcomes observed in our cohort, including 18% functional independence and 52% mortality at 90 days, fall within the range reported in these previous studies and reinforce the notion that technical success does not necessarily translate into favorable clinical recovery in very elderly individuals. These findings emphasize that, in nonagenarians, technical success should be regarded as a necessary but not sufficient condition for favorable long-term outcomes, highlighting the importance of realistic prognostic counselling and careful patient selection.

An important aspect of our study is the emphasis on detailed pre-treatment clinical assessment. In contrast to routine decision-making in younger patients, evaluation of nonagenarians involved particularly careful determination of pre-stroke functional status, comorbidity burden, estimated life expectancy, neuroimaging findings, and anticipated recovery potential. Whenever necessary, information obtained from medical records was supplemented through structured interviews with family members or caregivers. Only patients with preserved pre-stroke independence, favorable imaging profiles, and no terminal comorbidities were considered eligible for thrombectomy. Although our study was not designed to evaluate this assessment strategy itself, the findings highlight the importance of individualized decision-making in very elderly patients. In routine clinical practice, chronological age frequently serves as a surrogate marker for frailty and biological vulnerability. However, substantial heterogeneity exists among nonagenarians, and selected individuals may retain sufficient functional reserve to derive meaningful benefit from reperfusion therapy. Our results therefore support a patient-centered approach in which eligibility for thrombectomy is guided by overall clinical profile rather than age alone.

The interpretation of between-group comparisons requires caution. The two cohorts were not designed to represent fully exchangeable populations. Nonagenarians were accrued over a longer period and underwent dedicated pre-treatment assessment, whereas the younger cohort represented contemporary routine practice. In addition, important baseline differences were present, particularly in pre-stroke mRS and chronic kidney disease. In unadjusted analyses, nonagenarians had markedly lower odds of functional independence and higher odds of mortality. After adjustment for pre-stroke mRS, admission NIHSS score, ASPECTS score, onset-to-groin puncture time, and chronic kidney disease, the estimated associations were substantially attenuated. These findings should not be interpreted as evidence that chronological age is unimportant or that outcomes are equivalent between groups. Rather, they suggest that part of the observed age-associated difference may be related to baseline functional vulnerability, stroke-related prognostic factors, and comorbidity burden. The wide confidence intervals indicate that a clinically relevant residual association with age cannot be excluded. Sensitivity analyses based on propensity score overlap weighting yielded qualitatively similar effect directions, although the magnitude of the estimated associations differed to some extent. These findings highlight the influence of the target population and the remaining uncertainty related to limited covariate overlap between age groups. Accordingly, the purpose of multivariable adjustment was to better understand the contribution of baseline prognostic factors to the observed between-group differences, rather than to determine whether chronological age itself was clinically relevant.

Regarding safety, parenchymal hematoma occurred more frequently among nonagenarians, and this association persisted after adjustment for admission NIHSS score and intravenous thrombolysis. The higher frequency of parenchymal hematoma may reflect increased vascular fragility and reduced cerebrovascular reserve in very elderly patients, although this observation should be interpreted cautiously given the limited sample size. In contrast, rates of symptomatic intracerebral hemorrhage were comparable between age groups regardless of the definition applied. These observations are consistent with previous reports suggesting that advanced age does not necessarily confer a markedly increased risk of clinically significant hemorrhagic complications following thrombectomy [16].

The present study has several strengths. It includes a consecutive cohort of nonagenarians treated in routine clinical practice at a high-volume comprehensive stroke center. In addition, it provides a detailed description of the structured pre-treatment clinical assessment applied to very elderly patients considered for MT.

Several limitations should be acknowledged. First, this was a retrospective single-center study, which may limit the generalizability of the findings and introduces the possibility of selection bias. Second, nonagenarians were accrued over a longer time period than the younger comparator cohort, potentially introducing secular bias related to evolving thrombectomy techniques, workflow improvements, and changes in stroke care organization. Third, the detailed pre-treatment clinical assessment applied to nonagenarians resulted in a highly selected study population with preserved pre-stroke functional status and favorable clinical characteristics; therefore, the findings should not be generalized to all patients aged ≥ 90 years presenting with acute ischemic stroke. Fourth, residual confounding due to unmeasured factors cannot be excluded. In particular, objective frailty measures, cognitive status, collateral circulation quality, and other markers of biological reserve were not systematically available. Fifth, the limited sample size constrained the number of covariates that could be included in multivariable models and resulted in imprecise estimates for several outcomes. In addition, pre-stroke disability and chronic kidney disease may partly reflect biological vulnerability accumulated with advancing age. Adjustment for these variables may therefore have accounted for part of the total age-associated difference in outcomes and attenuated the estimated age-group association. Accordingly, the adjusted estimates should be interpreted as associations conditional on selected baseline clinical characteristics rather than as estimates of the total effect of chronological age. Finally, the propensity score overlap-weighted analysis was restricted by limited covariate overlap between groups and should be interpreted as exploratory rather than definitive.

## Conclusions

MT achieved high rates of successful reperfusion in carefully selected nonagenarians treated at an experienced comprehensive stroke center. Although functional outcomes and mortality remained less favorable than in younger patients, adjustment for baseline clinical and prognostic factors attenuated the estimated associations without excluding a clinically relevant effect of age. These findings support cautious, individualized treatment decisions based on chronological age together with pre-stroke function, comorbidity burden, imaging findings, and overall clinical profile.

## Data Availability

The data examined in this research is governed by the following licenses and restrictions: Due to privacy and ethical considerations, the data is not available to the public. To request access to these datasets, please contact bartosz.jablonski@gumed.edu.pl.
